# Nosocomial infections by *Klebsiella pneumoniae* carbapenemase producing enterobacteria in a teaching hospital

**DOI:** 10.1590/S1679-45082014AO3131

**Published:** 2014

**Authors:** Gabriela Seibert, Rosmari Hörner, Bettina Holzschuh Meneghetti, Roselene Alves Righi, Nara Lucia Frasson Dal Forno, Adenilde Salla

**Affiliations:** 1 Universidade Federal de Santa Maria, Santa Maria, RS, Brazil.

**Keywords:** Drug resistance, Carbapenems/isolation & purification, Enterobacteriaceae, Cross infection/prevention & control, Klebsiella pneumoniae/epidemiology

## Abstract

**Objective:**

To analyze the profile of patients with microorganisms resistant to carbapenems, and the prevalence of the enzyme *Klebsiella pneumoniae *carbapenemase in *interobacteriaceae*.

**Methods:**

Retrospective descriptive study. From the isolation in bacteriological tests ordered by clinicians, we described the clinical and epidemiological characteristics of patients with enterobacteria resistants to carbapenems at a university hospital, between March and October 2013.

**Results:**

We included 47 isolated patients in this study, all exhibiting resistance to carbapenems, including 9 patients who were confirmed as infected/colonized with *K. pneumoniae *carbapenemase. Isolation in tracheal aspirates (12; 25.5%) predominated. The resistance to ertapenem, meropenem, and imipenem was 91.5%, 83.0% and 80.0%, respectively. Aminoglycosides was the class of antimicrobials that showed the highest sensitivity, 91.5% being sensitive to amikacin and 57.4% to gentamicin.

**Conclusion:**

The *K. pneumoniae* carbapenemase was an important agent in graun isotaling in hospital intection. The limited therapeutic options emphasize the need for rapid laboratory detection, as well as the implementation of measures to prevent and control the spread of these pathogens.

## INTRODUCTION

Bacterial resistance is a frequent and significant problem in the hospital environment. Increased resistance among members of the *Enterobaceriaceae* family have culminated in the ever more frequent appearance of multiresistant species, which represent an important public health problem that is in expansion, requiring multidisciplinary efforts for prevention and control, besides efficient laboratorial detection.^(1, 2)^

Among the *Gram*-negative bacteria, the production of beta-lactamases is the primary form of bacterial resistance to betalactamic antimicrobials. Betalactamases are enzymes that promote degradation of the betalactamic ring, inactivating the antimicrobial and impeding its activity against the enzymes responsible for synthesis of the bacterial cell wall. Among the betalactamases, currently the most concerning groups are the amplified aspect betalactamases and the carbapenemases.^(3)^

Carbapenemases are more frequently found in enterobacteria, and predominate in the *Klebsiella*, *Enterobacter*, *Escherichia*, *Serratia*, *Citrobacter*, *Salmonella*, *Proteus, *and *Morganella *genera*.* ^(4)^ The most prevalent carbapenemases in enterobacteria are coded by genes from the *bla*KPC, *bla*IMP, *bla*VIM, *bla*Ndm and *bla*Oxa groups,^(5)^ among which the production of *Klebsiella pneumoniae *carbapenemase (KPC) has become an emergent mechanism.

KPC is a betalactamase belonging to Ambler’s Class A, and Bush’s subgroup 2f.^(6)^ This enzyme confers resistance to all betalactamic agents such as cephalosporins, penicillins, monobactams, and carbapenems. The latter class of antibiotics is wide spectrum, frequently used in treating infections caused by multiresistant bacteria. Therefore, for the treatment of KPC-producing microorganisms, there are few therapeutic options left. This characteristic, along with the fact of KPC having a high potential for dissemination due to its plasmid location, which facilitates transfer to the interspecies gene, has been reason for concern in hospitals and healthcare institutions in all parts of the world.^(7)^

An ideal phenotypic methodology for KPC identification has not yet been described, and those currently available have low specificity, making *bla*KPC gene investigation necessary for the confirmation of the resistance mechanism.^(8)^

If the resistance to carbapenems is confirmed, the current recommendation of the *Agência Nacional de Vigilância Sanitária* (ANVISA), in Technical Note 01/2013, consists in performing enzyme inhibition tests with the combined use of specific beta-lactamase inhibitors such as phenylboronic acid (FBA), cloxacillin, and ethylenediaminetetraacetic acid (EDTA). However, these phenotypic tests are basically triage since only the molecular tests, such as the polymerase chancing reaction (PCR) and sequencing, are confirmatory.^(9)^

Early detection of patients infected or colonized by KPC is of great importance, since these microorganisms may cause severe infections. Additionally, there is a need for implantation of adequate precautions in contact and training of these patients, thus providing control of dissemination of this type of resistant mechanism in Brazil and the world.^(10)^

Due to this great dissemination of multiresistant enterobacteria over the last few years, the objective of the present study was to describe the epidemiological profile of the patients seen at a university hospital who presented with carbapenem-resistant enterobacteria, determining age, gender, area of hospitalization, species isolated, and clinical specimen of the test, as well as the sensitivity profile of the clinical isolates.

## OBJECTIVE

To analyze the profile of patients who presented with carbapenem-resistant microorganisms, and the prevalence of the *Klebsiella pneumoniae *carbapenemase enzyme in enterobacteria.

## METHODS

In February 2013, the first KPC was identified at *Hospital Universitário de Santa Maria* (HUSM), a teaching hospital with about 328 beds where this research was carried out. As of March that year, we initiated this retrospective descriptive study by selecting all of the samples identified as suspect KPC-producers by the Microbiology Laboratory of the Institution, using phenotypic automatized phenotypic (Vitek 2^®^– bioMérieux) and/or manual methodology.

Between March and October 2013, 47 nosocomial isolates were obtained from enterobacteria with reduced sensitivity to carbapenems ertapenem, imipenem, and meropenem. The samples were from various clinical specimens (urine, feces, tracheal aspirate, blood, and catheter).

A retrospective observational study of the sensitivity to antibiotics was conducted, considering the resistance to third-generation cephalosporins (ceftazidime, ceftriaxone, or cefotaxime), fourth-generation cephalosporins (cefepime), carbapenems (imipenem, meropenem, or ertapenem), aminoglycosides (gentamicin or amikacin), and tigecycline.

Posteriorly, an analysis of the profile of patients colonized/infected by these enterobacteria was performed, observing the clinical specimens of isolated strains, hospital unit, age, and gender of the patients seen at the HUSM.

The data were collected from medical reports and/or based on computed hospital data.

All the samples were sent to the *Laboratório Central do Estado* (LACEN) to investigate the *bla*KPC gene using molecular biology (PCR).

The present study was approved by the Ethics in Research Committee under registration #10291913. 3.0000.5346, with the respective Confidentiality Agreement.

## RESULTS

**Figure 1:**
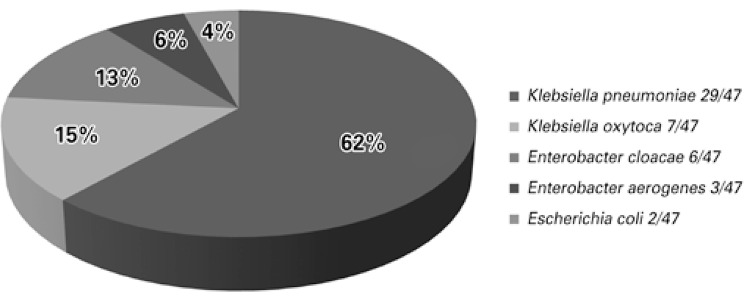
Distribution of the carbapenem-resistant enterobacteria

During the period analyzed, 47 isolates were identified suspected of being KPC-producers. Figure 1 shows the microorganisms isolated.

As to the gender of the patients, there was a predominance of males (34; 72.3%). The hospital units where the microorganisms were isolated are thus distributed: 14 (29.8%) in surgical clinic, 12 (25.6%) in the intensive care units (ICU), 8 (17.0%) in the adult emergency care unit (ER), 7 (14.9%) in the gynecology and obstetrics clinic, 5 (10.6%) in the outpatient clinic, and 1 (2.1%) at the *Centro de Tratamento da Criança e do Adolescente com Câncer.*

The age ranges for isolation were from zero to 10 years for 4 (8.5%) samples, from 11 to 60 years for 16 (34.0%), and >60 years for 27 (57.5%).

**Figure 2:**
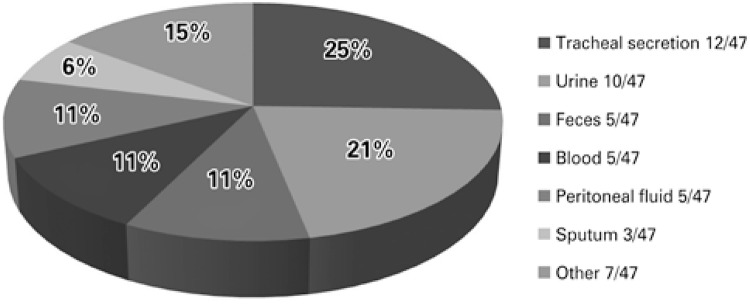
Distribution according to the clinical specimen of the isolated enterobacteria under study

As to the isolated clinical specimen, the greatest number occurred from tracheal secretions (12; 25%), followed by urine, blood culture, feces, and peritoneal fluid, as is represented in figure 2.

The sensitivity profile to carbapenems (imipenem, ertapenem, and meropenem), cephalosporins (ceftriaxone and cefepime), aminoglycosides (amikacin and gentamicin), and tigecycline is seen on table 1. For interpretation of the sensitivity tests, the criteria standardized in ANVISA’s Technical Note 01/2013^(9)^ and *Manual of Clinical and Laboratory Standards Institute* (CLSI) 2013^(11)^ were used.

**Table 1:** Sensitivity profile of the isolated enterobacteria

Antimicrobial	Sensitive n (%)	Intermediary n (%)	Resistant n (%)	Total tested
Amikacin	43 (91.5)	0 (0)	4 (8.5)	47
Gentamycin	27 (57.4)	0 (0)	20 (42.6)	47
Tigecycline	25(69.4)	5 (13.9)	6 (16.7)	36
Ceftriaxone	13 (27.6)	3 (6.4)	31 (66.0)	47
Cefepime	15 (31.9)	6 (12.8)	26 (55.3)	47
Ertapenem	3 (6.4)	1 (2.1)	43 (91.5)	47
Imipenem	7 (17.5)	1 (2.5)	32 (80.0)	40
Meropenem	8 (17.0)	0 (0)	39 (83.0)	47

Of the total 47 isolates, 9 had confirmation of the *bla*KPC gene by PCR. Four of the patients in whom the KPC-producing microorganism was identified died.

## DISCUSSION

Currently, the dissemination of KPC-producing enterobacteria is a serious clinical and epidemiological problem in various healthcare institutions in Brazil.^(9)^ Therefore, it is extremely relevant to know the local epidemiological standards and the sensitivity profile by means of methodologies applicable in any clinical microbiology laboratory for detection of strains that produce carbapenemases and thus, to contribute towards the reduction of indices of morbidity and mortality.

The term, “KPC”, is associated with the bacterial species in which the enzyme was found for the first time in 1996, in North Carolina, in a case of *K. pneumoniae.* ^(12)^ This enzyme has already been identified in practically all members with clinical importance of the *Enterobacteriaceae* family, but it occurs more frequently in *K. pneumoniae.* ^(13)^

In the present study, *K. pneumoniae *was the microorganism that presented with the greatest resistance to carbapenems (29; 62.0%), followed by *Enterobacter* sp. (9; 19.1%). These results are in agreement with the study conducted by Almeida et al.^(14)^ in the city of São Paulo (SP), in which, from 40 samples that presented with the *bla*KPC gene (PCR), 38 were *K. pneumoniae *and 2 were *Enterobacter cloacae*. Still in this study, the authors found that the isolates originated from different clinical specimens such as urine, tracheal secretion, blood culture, pancreatic abscess secretion, and the largest isolation occurred in a rectal swab, representing 42.5%,^(14)^ different from the present study in which the greatest isolation was in tracheal aspirates (12; 25%), followed by urine (10; 21%).

The hospital unit where the greatest occurrence in our study was found was the surgical clinic (14; 29.8%), followed by the ICU. These data are in agreement with the study performed by Alves and Behar, in which the greatest isolation also occurred in the surgical clinic (25/77; 32%), followed by patients hospitalized in clinical and internal medicine specialties (24/77; 31%) and ICU (23/77; 29%).^(3)^

Infections caused by KPC-producing microorganisms generally affect immunosuppressed patients who are hospitalized and/or who use invasive devices, such as catheters and tubes.^(15)^

The age range in which there was greatest isolation of carbapenem-resistant enterobacteria in our study was more than 60 years of age (27; 57.5%), similar to a study done by Bradford et al., in which the mean age of patients with KPC-producing strains was 73 years.^(16)^ This fact also agrees with the study performed by Alves and Behar, in which the mean age was 60 years.^(3)^

As to gender, in the present study, there was male predominance (34; 72.3%), in agreement with the research carried out in Porto Alegre (RS) by Alves and Behar, in which the predominance of patients infected/colonized by KPC-producing enterobacteria also was of the male gender (47/77; 61%).^(3)^

In general, the isolates in our study presented a profile of multi-resistance to the antibiotics as per criteria covered in ANVISA’s Technical Note 01/2013, recording high resistance to carbapenems, to third-generation cephalosporins (66.0% resistant to ceftriaxone), and to fourth-generation cephalosporins (55.3% resistant to cefepime).

The low sensitivity to carbapenems found in our study is a worrisome matter. Our isolates were 80.0% resistant to imipenem, 83.0% to meropenem, and 91.5% to ertapenem. Ertapenem constituted a marker of resistance to carbapenems, and could be directly related to the KPC enzyme or to other mechanisms that decrease sensitivity to these antimicrobials in a specific way, such as with the production of other beta-lactamases and loss of purines.^(14)^ In the study by Bratu et al., which involved 62 isolates of *K. pneumoniae* that produced KPC, the resistance to carbapenems imipenem, meropenem, and ertapnenem was 98%, 96%, and 100%, respectively. In this study, the authors concluded that the resistance to ertapenem represented the most sensitive clinical test for detection of KPC production.^(17)^

Our study showed that antibiotics from the aminoglycoside group were those that presented with the greatest sensitivity in the isolates resistant to carbapenems. Amikacin showed greatest sensitivity (91.5%), followed by gentamicin (57.4%). In a study by Alves and Behar at *Hospital Nossa Senhora da Conceição* (HNSC) in Porto Alegre, a sensitivity of 97.5% was obtained for amikacin and 70% for gentamicin in isolates of *K. pneumoniae *that produced KPC.^(3)^ In another similar study carried out by Soares, 100% sensitivity was noted for gentamicin and 62.5% sensitivity for amikacin among the isolates of *K. pneumoniae *that produced KPC.^(18)^ Although the percentage values of this study differ from ours, the class of aminoglycosides continues as a good alternative for carbapenem-resistant microorganisms.

Tigecycline also demonstrated high sensitivity in our study, showing 69.4% (25/36) sensitivity and was in accordance with the study performed by Alves and Behar, in which, for this antibiotic, 39 samples of KPC-producing samples of *K. pneumoniae *were tested and of these, 31 (79.4%) displayed sensitivity.^(3)^ We observed good sensitivity to tigecycline and to the aminoglycosides, as these are a good alternative for treating enterobacteria resistant to carbapenems. However, we need to point out that amikacin, gentamicin, and tigecycline do not have adequate action in serious systemic infections; in these, an association of one or more antibiotics is indicated.^(3)^

As per ANVISA’s Technical Note 01/2013, appropriate empirical therapy for infections caused by multiresistant enterobacteria is the use of polymyxin B or polymyxin E (colistin), in association with one or more antibiotics such as aminoglycosides (gentamicin or amikacin), carbapenems (meropenem or doripenem), and tigecycline, avoiding the use of monotherapy due to the risk of developing resistance.^(9)^

Our study evinced an 8.5% mortality rate (n=4) in patients infected/colonized by carbapenem-resistant enterobacteria. In a study performed in Porto Alegre, the mortality rate related to the infection by KPC was 18%^(3)^.

Of the 47 isolates in the present study, only in 9 did we have confirmation of the *bla*KPC gene by PCR. In the other isolates, the resistance to carbapenems noted may be attributed to the presence of another resistance mechanism.

## CONCLUSION

Considering the high resistance to antibiotics and the great power of dissemination, KPC constitutes an important hospital pathogen, currently in growing isolation in hospitals of the entire world. The quick laboratorial detection of this resistance mechanism is vital and necessary, as well as the adoption of rigorous measures of dissemination prevention and control, and the implementation of precautions regarding contact and adequate treatment.

The profile found of the patient with suspected KPC in this study is that of a male over 60 years of age, hospitalized in the surgical clinic with isolation of the *Klebsiella pneumoniae* microorganism from tracheal secretion, and this microorganism displayed a high level of resistance to carbapenems.

Ertapenem proved to be the best indicator of resistance to carbapenems, and may or may not be related to the production of the *Klebsiella pneumoniae *carbapenemase enzyme.

Aminoglycosides and tigecycline displayed a good percentage of sensitivity, proving to be a reasonable therapeutic option in treating enterobacteria resistant to carbapenems; its association with one or more antibiotics is recommended.
