# Supplementary material for: Nosocomial infections by Klebsiella pneumoniae carbapenemase producing enterobacteria in a teaching hospital
Source: Einstein (Sao Paulo). 2014 Jul-Sep;12(3):282–6. doi: 10.1590/S1679-45082014AO3131 (PMC4872936; doi:10.1590/S1679-45082014AO3131)
Supplement: Translation

# Translation: Infecções hospitalares por enterobactérias produtoras de *Klebsiella pneumoniae *carbapenemase em um hospital escola

**Authors:** Gabriela Seibert, Rosmari Hörner, Bettina Holzschuh Meneghetti, Roselene Alves Righi, Nara Lucia Frasson Dal Forno, Adenilde Salla

**Affiliations:** 1 Universidade Federal de Santa Maria, Santa Maria, RS, Brasil.

**Keywords:** Resistência a medicamentos, Carbapenêmicos/isolamento & purificação, Enterobacteriaceae, Infecção hospitalar/prevenção & controle, Klebsiella pneumoniae/epidemiologia

## Abstract

**Objetivo:**

Analisar o perfil dos pacientes que apresentaram microrganismos com resistência aos carbapenêmicos, e a prevalência da enzima *Klebsiella pneumoniae *carbapenemase em enterobactérias.

**Métodos:**

Estudo retrospectivo descritivo. A partir do isolamento em exames bacteriológicos solicitados pelos clínicos, descrevemos as características clínicas e epidemiológicas dos pacientes que apresentaram enterobactérias resistentes aos carbapenêmicos entre março e outubro de 2013 em um hospital universitário.

**Resultados:**

Foram incluídos 47 pacientes isolados, todos apresentando resistência aos carbapenêmicos, dos quais 9 tiveram confirmação de infecção/colonização por *K. pneumoniae *carbapenemase. Ocorreu predomínio de isolamento em aspirados traqueais (12; 25,5%). A resistência ao ertapenem, meropenem e imipenem foi de 91,5%, 83,0% e 80,0%, respectivamente. Os aminoglicosídeos foram a classe de antimicrobianos que apresentou maior sensibilidade, sendo 91,5% sensível a amicacina e 57,4% a gentamicina.

**Conclusão:**

A *K. pneumoniae *carbapenemase constituiu um importante patógeno hospitalar em isolamento crescente nesse nosocômio. As limitadas opções terapêuticas reforçam a necessidade de uma rápida detecção laboratorial, assim como a implementação de medidas de prevenção e controle da disseminação desses patógenos.

## INTRODUÇÃO

A resistência bacteriana é um problema frequente e importante no ambiente hospitalar. O aumento da resistência entre os membros da família *Enterobaceriaceae* tem culminado no aparecimento cada vez mais frequente de espécies multirresistentes, as quais representam um importante problema de saúde pública em expansão, exigindo esforço multidisciplinar para prevenção e controle, além de uma detecção laboratorial eficiente.^(1, 2)^

Entre as bactérias *Gram*-negativas, a produção de betalactamases é a principal forma de resistência bacteriana aos antimicrobianos betalactâmicos. Betalactamases são enzimas que promovem a degradação do anel betalactâmico, inativando o antimicrobiano e impedindo que ele apresente atividade contra as enzimas responsáveis pela síntese da parede celular bacteriana. Entre as betalactamases, os grupos mais preocupantes, atualmente, são as betalactamases de aspecto ampliado e as carbapenemases.^(3)^

As carbapenemases ocorrem mais frequentemente em enterobactérias, sendo predominantes nos gêneros *Klebsiella*, *Enterobacter*, *Escherichia*, *Serratia*, *Citrobacter*, *Salmonella*, *Proteus* e *Morganella.* ^(4)^ As carbapenemases mais prevalentes em enterobactérias são codificadas por genes dos grupos *bla*KPC, *bla*IMP, *bla*VIM, *bla*Ndm e *bla*Oxa,^(5)^ entre as quais a produção de *Klebsiella pneumoniae *carbapenemase (KPC) tem se tornado um mecanismo emergente.

KPC é uma betalactamase pertencente à classe A de Ambler e ao subgrupo 2f de Bush.^(6)^ Essa enzima confere resistência a todos os agentes betalactâmicos como cefalosporinas, penicilinas, monobactâmicos e, inclusive, a carbapenêmicos. Essa última classe de antimicrobianos é de amplo espectro, com uso frequente no tratamento de infecções causadas por bactérias multirresistentes. Desse modo, para o tratamento de microrganismos produtores de KPC, restam escassas opções terapêuticas. Essa característica, juntamente do fato da KPC ter alto potencial de disseminação, devido à sua localização plasmidial, a qual facilita a transferência do gene interespécies, tem sido motivo de preocupação em hospitais e instituições de saúde em todo o mundo.^(7)^

Uma metodologia fenotípica ideal para identificação da KPC ainda não foi descrita, sendo que as que temos disponíveis apresentam pouca especificidade, fazendo-se necessária a pesquisa do gene *bla*KPC para a confirmação do mecanismo de resistência.^(8)^

Se comprovada a resistência aos carbapenêmicos, a recomendação atual da Agência Nacional de Vigilância Sanitária (ANVISA), em sua Nota Técnica 01/2013, consiste na realização de testes de inibição enzimática, com a utilização combinada de inibidores específicos de betalactamases, como o ácido fenilborônico (AFB), a cloxacillina e o ácido etilenodiaminotetracético (EDTA). Entretanto, esses testes fenotípicos consistem em uma triagem, pois apenas os testes moleculares, como a reação em cadeia da polimerase (PCR) e sequenciamento, são confirmatórios.^(9)^

A detecção precoce de pacientes infectados ou colonizados pela KPC é de grande importância, uma vez que esses microrganismos podem causar infecções graves. Além disso, é necessário que sejam implementadas precauções de contato e tratamento adequado desses pacientes, proporcionando, assim, o controle da disseminação desse tipo de mecanismo de resistência no Brasil e no mundo.^(10)^

Devido a essa grande disseminação de enterobactérias multirresistentes nos últimos anos, o objetivo do presente estudo foi descrever o perfil epidemiológico dos pacientes atendidos em um hospital universitário que apresentaram enterobactérias com resistência aos carbapenêmicos, determinando idade, sexo, área de internação, espécie isolada e espécime clínico do exame, assim como o perfil de sensibilidade dos isolados clínicos.

## OBJETIVO

Analisar o perfil dos pacientes que apresentaram microrganismos com resistência aos carbapenêmicos, e a prevalência da enzima *Klebsiella pneumoniae *carbapenemase em enterobactérias.

## MÉTODOS

Em fevereiro de 2013, foi identificada a primeira KPC no Hospital Universitário de Santa Maria (HUSM), um hospital escola com cerca de 328 leitos onde esta pesquisa foi desenvolvida. A partir de março daquele ano, iniciamos este estudo retrospectivo descritivo selecionando todas as amostras identificadas como suspeitas produtoras de KPC pelo Laboratório de Microbiologia da instituição, usando metodologia fenotípica automatizada (Vitek 2^®^– bioMérieux) e/ou manual.

Entre março e outubro de 2013, foram obtidos 47 isolados nosocomiais de enterobactérias com sensibilidade diminuída aos carbapenêmicos ertapenem, imipenem e meropenem. As amostras foram oriundas de diversos espécimes clínicos (urina, fezes, aspirado traqueal, sangue e cateter).

Foi realizado um estudo observacional retrospectivo da sensibilidade aos antimicrobianos, frente à resistência a cefalosporinas de terceira geração (ceftazidima, ceftriaxona ou cefotaxima), cefalosporina de quarta geração (cefepime), carbapenêmicos (imipenem, meropenem ou ertapenem), aminoglicosídeos (gentamicina ou amicacina) e tigeciclina.

Posteriormente, foi realizada uma análise do perfil dos pacientes colonizados/infectados por essas enterobactérias, sendo observados espécime clínico de isolamento das cepas, unidade hospitalar, idade e sexo dos pacientes atendidos no HUSM.

Os dados foram coletados a partir de prontuários médicos e/ou na base de dados hospitalar informatizada.

Todas as amostras foram encaminhadas ao Laboratório Central do Estado (LACEN) para a pesquisa do gene *bla*KPC por biologia molecular (PCR).

O presente estudo foi aprovado pelo Comitê de Ética em Pesquisa sob o registro 10291913.3.0000.5346, com o respectivo Termo de Confidencialidade.

## RESULTADOS

No período analisado, foram identificados 47 isolados suspeitos produtores de KPC. Na figura 1, encontram-se os microrganismos isolados.

**Figura 1:** Distribuição de enterobactérias isoladas com resistência aos carbapenêmicos

Em relação ao sexo dos pacientes, houve predomínio do masculino (34; 72,3%). As unidades hospitalares em que foram isolados os microrganismos estão assim distribuídas: 14 (29,8%) na clínica cirúrgica, 12 (25,6%) nas unidades de terapia intensiva (UTI), 8 (17,0%) no pronto atendimento (PA) adulto, 7 (14,9%) na clínica de ginecologia e obstetrícia, 5 (10,6%) no ambulatório e 1 (2,1%) no Centro de Tratamento da Criança e do Adolescente com Câncer.

As faixas etárias de isolamento foram de zero a 10 anos para 4 (8,5%) amostras, de 11 a 60 anos para 16 (34,0%) e >60 anos para 27 (57,5%).

Com relação a espécime clínico de isolamento, o maior número ocorreu das secreções traqueais (12; 25%), seguidas de urina, hemocultura, fezes e líquido peritoneal, como representado na figura 2.

**Figura 2:** Distribuição conforme espécime clínico de isolamento das enterobactérias em estudo

O perfil de sensibilidade aos carbapenêmicos (imipenem, ertapenem e meropenem), as cefalosporinas (ceftriaxona e cefepime), aos aminoglicosídeos (amicacina e gentamicina) e a tigeciclina encontra-se na tabela 1. Para a interpretação dos testes de sensibilidade, foram utilizados os critérios preconizados na Nota Técnica 01/2013 da ANVISA^(9)^ e *Manual Clinical and Laboratory Standards Institute* (CLSI) 2013.^(11)^

**Tabela 1:** Perfil de sensibilidade das enterobactérias isoladas

Antimicrobiano	Sensível n (%)	Intermediário n (%)	Resistente n (%)	Total testados
Amicacina	43 (91,5)	0 (0)	4 (8,5)	47
Gentamicina	27 (57,4)	0 (0)	20 (42,6)	47
Tigeciclina	25(69,4)	5 (13,9)	6 (16,7)	36
Ceftriaxona	13 (27,6)	3 (6,4)	31 (66,0)	47
Cefepima	15 (31,9)	6 (12,8)	26 (55,3)	47
Ertapenem	3 (6,4)	1 (2,1)	43 (91,5)	47
Imipenem	7 (17,5)	1 (2,5)	32 (80,0)	40
Meropenem	8 (17,0)	0 (0)	39 (83,0)	47

Do total de 47 isolados, 9 obtiveram confirmação do gene *bla*KPC por PCR. Quatro dos pacientes em que o microrganismo produtor de KPC foi identificado evoluíram a óbito.

## DISCUSSÃO

Atualmente, a disseminação de enterobactérias produtoras de KPC constitui um grave problema clínico e epidemiológico em várias instituições de saúde do Brasil.^(9)^ Dessa forma, é extremamente relevante conhecer os padrões epidemiológicos locais e o perfil de sensibilidade, por meio de metodologias aplicáveis a qualquer laboratório de microbiologia clínica, para detecção de cepas produtoras de carbapenemases e, assim, contribuir para redução dos índices de morbidade e mortalidade.

O termo “KPC” está associado à espécie bacteriana em que a enzima foi encontrada pela primeira vez, em 1996, na Carolina do Norte, em uma *K. pneumoniae.* ^(12)^ Essa enzima já foi identificada em praticamente todos os membros de importância clínica da família *Enterobacteriaceae*, porém ocorre mais frequentemente em *K. pneumoniae.* ^(13)^

No presente estudo, *K. pneumoniae *foi o microrganismo que apresentou maior resistência aos carbapenêmicos (29; 62,0%), seguida de *Enterobacter* sp. (9; 19,1%). Esses resultados estão de acordo com o estudo realizado por Almeida et al.,^(14)^ na cidade de São Paulo (SP), no qual, de 40 amostras que apresentaram o gene *bla*KPC (PCR), 38 eram *K. pneumoniae *e 2 *Enterobacter cloacae*. Ainda nesse estudo, os autores constataram que os isolados foram oriundos de diferentes espécimes clínicos, como urina, secreção traqueal, hemocultura, secreção do abscesso pancreático e o maior isolamento ocorreu em swab retal, representando 42,5%,^(14)^ diferentemente do presente estudo, em que o maior isolamento ocorreu dos aspirados traqueais (12; 25%), seguidos de urina (10; 21%).

A unidade hospitalar em que ocorreu a maior incidência em nossa pesquisa foi na clínica cirúrgica (14; 29,8%), seguida da UTI. Esses dados estão de acordo com o estudo realizado por Alves e Behar, segundo o qual o maior isolamento também ocorreu na clínica cirúrgica (25/77; 32%), seguido de pacientes internados nas especialidades clínicas e medicina interna (24/77; 31%) e UTI (23/77; 29%).^(3)^

Infecções causadas por microrganismos produtores de KPC geralmente acometem pacientes imunodeprimidos, hospitalizados e/ou que fazem uso de dispositivos invasivos, como cateter e sonda.^(15)^

A faixa etária em que houve o maior isolamento das enterobactérias resistentes aos carbapenêmicos, em nosso estudo, foi acima de 60 anos (27; 57,5%), similarmente a estudo realizado por Bradford et al., no qual a idade média dos pacientes portadores de cepas produtoras de KPC foi de 73 anos.^(16)^ Esse dado também concorda com o estudo realizando por Alves e Behar, no qual a média de idade foi de 60 anos.^(3)^

Em relação ao sexo, no presente estudo, houve predomínio do masculino (34; 72,3%), concordando com pesquisa realizada em Porto Alegre (RS) por Alves e Behar, na qual o predomínio de pacientes infectados/colonizados por enterobactérias produtoras de KPC também ocorreu no gênero masculino (47/77; 61%).^(3)^

Em geral, os isolados da nossa pesquisa apresentaram perfil de multirresistência aos antimicrobianos conforme critérios abordados na Nota Técnica 01/2013 da ANVISA, registrando alta resistência aos carbapenêmicos, às cefalosporinas de terceira geração (66,0% resistentes a ceftriaxona) e a cefalosporina de quarta geração (55,3% resistentes a cefepima).

A baixa sensibilidade aos carbapenêmicos encontrada em nosso estudo é preocupante. Nossos isolados apresentaram-se 80,0% resistentes a imipenem, 83,0% ao meropenem e 91,5% ao ertapenem. O ertapenem constitui um marcador de resistência aos carbapenêmicos, podendo estar relacionado diretamente à enzima KPC ou a outros mecanismos, que diminuem a sensibilidade a esse antimicrobiano de forma específica, como produção de outras betalactamases e perda de porinas.^(14)^ Na pesquisa realizada por Bratu et al., a qual envolveu 62 isolados de *K. pneumoniae* produtoras de KPC, a resistência aos carbapenêmicos imipenem, meropenem e ertapnenem foi de 98%, 96% e 100%, respectivamente. Nesse estudo, os autores concluíram que a resistência ao ertapenem representou o teste clínico mais sensível para detecção da produção de KPC.^(17)^

Nosso estudo demonstrou que os antimicrobianos do grupo dos aminoglicosídeos foram os que apresentaram maior sensibilidade nos isolados resistentes aos carbapenêmicos. A amicacina apresentou maior sensibilidade (91,5%), seguida da gentamicina (57,4%). Em estudo realizado por Alves e Behar, no Hospital Nossa Senhora da Conceição (HNSC), em Porto Alegre, foi obtido sensibilidade de 97,5% para amicacina e de 70% para gentamicina em isolados *K. pneumoniae *produtoras de KPC.^(3)^ Em outro estudo semelhante realizado por Soares, foram observados 100% de sensibilidade a gentamicina e 62,5% de sensibilidade a amicacina entre isolados de *K. pneumoniae *produtores de KPC.^(18)^ Embora os valores percentuais desse estudo difiram do nosso, a classe dos aminoglicosídeos continuam sendo uma boa alternativa para microrganismos resistentes aos carbapenêmicos.

A tigeciclina também demonstrou alta sensibilidade em nosso estudo, apresentando 69,4% (25/36) de sensibilidade e estando de acordo com estudo realizado por Alves e Behar, no qual foram testadas, para esse antimicrobiano, 39 amostras de *K. pneumoniae *produtoras de KPC e, destas, 31 (79,4%) apresentaram sensibilidade.^(3)^ Podemos observar boa sensibilidade a tigeciclina e aos aminiglicosídeos, sendo estes uma boa alternativa para tratamento de enterobactérias resistentes aos carbapenêmicos. Porém, cabe ressaltar que a amicacina, a gentamicina e a tigeciclina não têm ação adequada em infecções sistêmicas graves, sendo indicada a associação de um ou mais antimicrobianos.^(3)^

De acordo com a Nota Técnica 01/2013 da ANVISA, a terapia empírica apropriada para infecções por enterobactérias multirresistentes é a utilização de polimixina B ou polimixina E (colistina), em associação com um ou mais antimicrobianos, como aminoglicosídeos (gentamicina ou amicacina), carbapenêmicos (meropenem ou doripenem) e tigiciclina, evitando-se a utilização de monoterapia, devido ao risco de desenvolvimento de resistência.^(9)^

Nossa pesquisa evidenciou uma taxa de mortalidade de 8,5% (n=4) nos pacientes infectados/colonizados por enterobactérias resistentes aos carbapenêmicos. Em estudo realizado em Porto Alegre, a taxa de mortalidade relacionada à infecção por KPC foi de 18%^(3)^.

Dos 47 isolados do presente estudo, apenas em 9 obtivemos confirmação do gene *bla*KPC por PCR. Nos demais isolados a resistência aos carbapenêmicos verificada pode ser atribuída à presença de outro mecanismo de resistência.

## CONCLUSÃO

Considerando a alta resistência aos antimicrobianos e o grande poder de disseminação, a KPC constitui um importante patógeno hospitalar, atualmente em isolamento crescente em nosocômios no mundo inteiro. Torna-se importante e necessária a rápida detecção laboratorial desse mecanismo de resistência, assim como a adoção de medidas rigorosas de prevenção e controle de disseminação, como a implementação de precauções de contato e tratamento adequado.

O perfil do paciente com suspeita de KPC encontrado no presente estudo é: homem, com mais de 60 anos, internando na clínica cirúrgica com isolamento do microrganismo *Klebsiella pneumoniae* da secreção traquel e esse microrganismo apresentando alta resistência aos carbapenemicos.

O ertapenem mostrou-se ser o melhor indicador de resistência aos carbapenêmicos, podendo estar ou não relacionado à produção da enzima *Klebsiella pneumoniae *carbapenemase.

Os aminoglicosídeos e a tigeciclina apresentaram um bom percentual de sensibilidade, mostrando-se uma razoável opção terapêutica no tratamento de enterobactérias resistentes aos carbapenêmicos e sendo recomendada sua associação com um ou mais antimicrobianos.
